# Thrombotic thrombocytopenic purpura in pregnancy: Lessons from a case series of three patients

**DOI:** 10.1177/03000605261421024

**Published:** 2026-03-19

**Authors:** Sarah A Elkourashy, Tamader Mashhadi, Amna Al-Kuwari, Sara Al-Abdulla, Gamal Sayed

**Affiliations:** 1 Department of Hematology and Bone Marrow Transplant, National Centre for Cancer Care and Research, Hamad Medical Corporation, Qatar; 2 Weill Cornell Medicine, Qatar; 3 Department of Obstetrics & Gynecology, Women’s Wellness and Research Center, Qatar; 4 Clinical Department, College of Medicine, 61780 Qatar University, Qatar

**Keywords:** Thrombotic thrombocytopenic purpura, A disintegrin and metalloprotease with thrombospondin type 1 motifs member 13, thrombocytopenia, therapeutic plasma exchange, pregnancy, caplacizumab, rituximab

## Abstract

Thrombotic thrombocytopenic purpura is a rare but life-threatening complication during pregnancy. Historically, maternal mortality exceeded 90% before the introduction of therapeutic plasma exchange, which remains the cornerstone of treatment. Rituximab has become increasingly valuable in managing refractory or relapsing disease, even during pregnancy, by reducing the risk of future episodes. In severe or recurrent cases, caplacizumab provides rapid control of acute thrombotic thrombocytopenic purpura by inhibiting platelet–von Willebrand factor interaction, although its use in pregnancy remains limited. Monitoring ADAMTS13 activity is essential to distinguish congenital from acquired thrombotic thrombocytopenic purpura, guide treatment decisions, and prevent relapses. This case series describes one acquired and two congenital thrombotic thrombocytopenic purpura cases in pregnancy, including a severe relapsing case successfully treated with caplacizumab. These cases underscore the importance of early diagnosis, individualized treatment, multidisciplinary care, and proactive management to optimize maternal and neonatal outcomes.

## Background

Thrombotic thrombocytopenic purpura (TTP) is a rare but serious complication in pregnancy, with an estimated incidence of 1 in 25,000 pregnancies.^1^ It results from a deficiency of the A disintegrin and metalloprotease with thrombospondin type 1 motifs member 13 (ADAMTS-13) enzyme,^2^ leading to the accumulation of ultra-large von Willebrand factor (vWF) multimers that promote platelet aggregation and microvascular thrombosis.^3^

Approximately half of the pregnancy-associated TTP cases present in the third trimester. Diagnosis is challenging because its clinical and laboratory features overlap with other pregnancy-related thrombotic microangiopathies (TMAs), including preeclampsia, hemolysis, elevated liver enzymes, and low platelet count (HELLP) syndrome, disseminated intravascular coagulation (DIC), and antiphospholipid syndrome.^4,5^ The wide spectrum of TTP presentations—from mild symptoms to life-threatening disease—requires a high index of suspicion, as untreated TTP carries a significant risk of maternal morbidity and mortality.^6^

Therapeutic plasma exchange (TPE) has dramatically reduced mortality and remains the cornerstone of treatment.^7^ Depending on disease severity and recurrence, additional therapies such as corticosteroids, rituximab,^8^ and the newer agent caplacizumab can be used to suppress the immune response and reduce microvascular thrombosis.^9^

This case series describes three pregnancy-related TTP cases managed at the Women’s Wellness and Research Center (WWRC) and Hamad General Hospital (HGH) in Doha, Qatar, Qatar, detailing their clinical presentations, treatment approaches, and the role of interdisciplinary collaboration in achieving favorable outcomes. All patients provided informed consent before receiving treatment. Our institution uses the latest-generation Spectra Optia system for TPE. Plasma volume is calculated based on the patient’s current body weight without adjustment. In all cases, 1–1.5 plasma volumes were exchanged, with an average procedure duration of 90–140 min.

## Case report 1

A 34-year-old gravida 7 para 6 woman at 33 weeks of gestation presented at the end of 2019 with headache, dysuria, dark-colored urine, and lower-limb swelling. She denied neurological symptoms other than headache. Her blood pressure was normal at 101/61 mmHg. Initial laboratory investigations showed thrombocytopenia (9 × 10^9^/L), normocytic normochromic anemia, elevated bilirubin and lactate dehydrogenase (LDH), a normal coagulation profile, normal kidney and liver function, and a peripheral smear suggestive of microangiopathic hemolytic anemia (MAHA) (Table 1).

**Table 1.** Summary of laboratory values in three cases before and after treatment.

Demography(Age, gravidity, GA)		Case 1		Case 2		Case 3	
		34Y/G7P6/33w		24Y/G1P0/31w		36Y/G4P2/37 + 4w	
Clinical presentation		New-onset headache and dark-colored urine		Sudden-onset inability to speak, right facial and limb weakness		Routine labs revealed low platelet count	
Lab value	Reference range	Initial values	After treatment	Initial values	After treatment	Initial values	After treatment
Hemoglobin (g/dL)	12.0–15.0	9.5	9	10.9	11.6	10.5	11.8
Platelets (×10^9^/L)	150–400	9	149	31	140	22	326
Prothrombin time (s)	9.7–11.8	10.1	10.3	11.6	10.3	9.8	10.5
INR	–	1.0	1.0	1.0	1.0	0.9	1.0
Urea (mmol/L)	2.76–8.07	3.60	2.6	2.6	4.2	7.3	5.6
Creatinine (µmol/L)	44–80	49	42	42	60	77	67
Bilirubin T (µmol/L)	0–21	28.0	4	9	7	10	3
LDH (U/L)	135–214	874	145	510	183	860	206
ALT (U/L)	0.0–33.0	9.4	20.8	8	9	11	13
AST (U/L)	0–32	28	14	18	13	34	11
Alk Phos (U/L)	35.0–104.0	114	58	77	68	143	49
ADAMTS13	ADAMTS13	36% (reference >70%)		5% (>70%)		<0.03 (Normal 0.68–1.63 IU/mL)	
	ADAMTS13 Inhibitor(normal)	Negative		Negative		0.8, Positive	
P.S.		Moderate normochromic normocytic anemia with anisocytosis, moderate polychromasia and moderate schistocytes. Mild left shift in leukocytes with marked thrombocytopenia		Moderate normocytic normochromic anemia with moderate schistocytes (3%), few polychromatophilic cells, and few microspherocytes. Leukocytosis with neutrophilia. Platelets markedly reduced.		Mild normocytic normochromic anemia with reticulocytosis, moderate schistocytes (7%), polychromasia, and few microspherocytes Leukocytosis with mild neutrophilia and left shift. Platelets markedly reduced.	

ALT: alanine aminotransferase; AST: aspartate aminotransferase; Alk Phos: alkaline phosphatase; LDH: lactate dehydrogenase; INR: international normalized ratio; GA: gestational age; G: gravidity; P: parity; Y: years; W, weeks; T: total; ADAMTS13: A disintegrin and metalloproteinase with thrombospondin motifs 13; P.S.: peripheral smear.

TTP was suspected, and the patient was started on high-dose intravenous dexamethasone (1 mg/kg, 40 mg daily for 4 days). TPE was initiated, exchanging 3.5–4 L of fresh frozen plasma via a right internal jugular central dialysis catheter. Each session included calcium gluconate (2 g in 100 mL normal saline) and premedication with intravenous diphenhydramine (25 mg). Plasma volume was adjusted to 1–1.5 times the patient’s estimated plasma volume.

The patient initially responded to TPE; however, platelet counts began to decline after 7 days. ADAMTS13 activity returned at 36% (reference range >70%), consistent with congenital TTP (cTTP), although results may have been elevated due to prior TPE.

On hospital day 10, the platelet count fell below 50 × 10^9^/L. She was started on dexamethasone pulse therapy (40 mg IV daily for 4 days) and a trial of intravenous immunoglobulin (30 g) to further improve her platelet counts. The platelet counts subsequently rose, accompanied by improvement in hemolysis.

On day 13, after 11 TPE sessions, induction of labor was initiated at 35 + 4 weeks following a multidisciplinary team decision due to declining platelet counts (66 × 10^9^/L) and plasmapheresis resistance. The platelet count increased to 118–123 × 10^9^/L with a stable hemoglobin; however, induction failed, and the patient underwent a lower segment cesarean section on day 15, delivering a healthy male infant. Two additional TPE sessions were administered postoperatively.

A complete workup, including computed tomography (CT) of the thorax, abdomen, and pelvis, combined with a negative autoimmune screen, excluded secondary causes such as malignancy.

The patient was discharged on postoperative day 8 on tapering prednisolone with a hemoglobin of 10.2 g/dL, hematocrit of 31%, and platelet count of 369 × 10^9^/L, after a total of 11 TPE sessions.

One year later, the patient conceived spontaneously and had an uncomplicated pregnancy. She was followed up regularly in the obstetric-hematology clinic in WWRC with serial complete blood counts and hemolytic markers, all of which remained normal. She delivered at 38 weeks via elective cesarean section.

**Figure 1.**
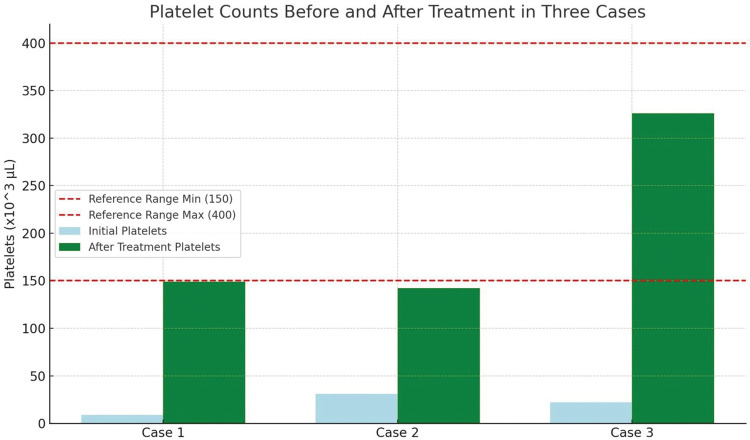
Platelet counts in three patients diagnosed with TTP before and after treatment. TTP: thrombotic thrombocytopenic purpura.

Figure 1 illustrates the platelet trends for all three patients before and after treatment.

## Case report 2

A 24-year-old primigravida at 31 weeks of gestation presented at the end of 2022 with sudden-onset speech difficulty and right-sided weakness, suggestive of an acute stroke. She denied additional symptoms except for transient numbness and paresthesia in her right upper limb 2 months ago, which resolved spontaneously. Her pregnancy had been uneventful, and she was normotensive on presentation.

Initial laboratory evaluation showed normocytic normochromic anemia (hemoglobin: 9.5 g/dL, hematocrit: 28.4%), thrombocytopenia with manual platelet counts of 81 × 10^9^/L, normal fibrinogen, normal coagulation profile (APTT/PT/INR), normal total bilirubin (7 µmol/L), normal liver function tests (aspartate aminotransferase (AST)/alanine aminotransferase (ALT)), and normal serum creatinine. The initial peripheral smear showed moderate normochromic normocytic anemia with a with no clear shistocytes schistocytes, raising suspicion for gestational thrombocytopenia (GT) or immune thrombocytopenia (ITP).

Brain magnetic resonance imaging confirmed a left middle cerebral artery infarct with suspected carotid dissection. As she presented outside the thrombolysis window, aspirin (100 mg daily) was initiated but later withheld when platelet counts dropped below 70 × 10^9^/L.

Repeat laboratory testing showed a significant drop in hemoglobin (8.4 g/dL), hematocrit (25.3%), and platelet count (37 × 10^9^/L), with increased schistocytes consistent with MAHA. Hemolytic markers were positive, with elevated LDH (514 U/L), low haptoglobin (<10 mg/dL), and a negative direct antiglobulin test (DAT). Autoimmune and thrombophilia screens were negative.

TTP was strongly suspected. TPE was promptly initiated with 3.5 L of plasma exchange daily, along with high-dose intravenous methylprednisolone (100 mg daily). Packed red blood cells were transfused to correct her anemia before TPE. Over the next 5 days, hemolysis parameters gradually improved, and platelet counts began to recover.

On day 6, the patient underwent a primary cesarean section under general anesthesia due to pregnancy-associated TTP complicated by ischemic stroke. Preoperative laboratory values showed a platelet count of 93 × 10^9^/L, normal coagulation studies, hemoglobin level of 10.2 g/dL, and hematocrit level of 30.1%. TPE was conducted on the day of surgery, and fresh frozen plasma (FFP) was administered. The delivery was uneventful. Postpartum, the patient required four additional TPE sessions.

The ADAMTS13 activity, measured before the initiation of TPE, was 5% (reference >70%) with no inhibitor, confirming a diagnosis of hereditary TTP. The patient required a total of seven TPE sessions before full hematological recovery. She was discharged on postoperative day 11 on oral prednisolone (120 mg daily) with a stable hematocrit of 31.1%, hemoglobin of 10.4 g/dL, and platelet count of 292 × 10^9^/L.

The neurology team recommended continuing aspirin (100 mg) for 6 months. She was then transferred to a rehabilitation center for further care. ADAMTS13 activity was repeated twice after delivery and remained below 10% (reference >70%) without inhibitors. Custom gene testing for the ADAMTS13 gene via next-generation sequencing was negative.

Follow-up visits with routine laboratory tests showed that the patient remained in remission.

## Case report 3

A 36-year-old gravida 4 para 2 woman at 37 + 4 weeks of gestation was admitted in mid-2022 with severe thrombocytopenia (22 × 10^9^/L) detected on routine testing. She had a history of low platelets in a previous pregnancy that resolved postpartum and a history of a transient ischemic attack (TIA) 3 months after delivery, for which she was maintained on low-dose aspirin but was not fully investigated. Her thrombophilia screen was negative except for heterozygous factor V Leiden mutation.

Her current pregnancy had been uneventful until admission, wherein she presented with hypertension (130/90 mmHg). Initial laboratory evaluation revealed normocytic normochromic anemia (hemoglobin: 10.5 g/dL, hematocrit: 29.3%), platelet count of 22 × 10^9^/L, elevated LDH (860 U/L), Low Haptoglobin <10 mg/dL, uric acid of 395 µmol/L, proteinuria (+2 on dipstick, urinary protein ratio of 26.31 mg/mmol), normal coagulation profile (APTT/PT/INR), normal total bilirubin, mildly elevated AST (34 U/L), normal ALT, and normal creatinine. Peripheral smear findings were compatible with MAHA.

The initial working diagnosis was preeclampsia with atypical HELLP syndrome versus TTP. A shared decision was made for an emergency cesarean section, which was performed on day 1 of hospital admission under general anesthesia.

Postoperatively, the patient’s platelet count continued to decline, and a diagnosis of TTP was made. TPE was immediately initiated after optimizing her hemoglobin via packed red blood cell transfusion. Daily TPE sessions continued for 1 week, during which platelet counts reached 194 × 10^9^/L. However, after stopping TPE, platelet counts dropped again to 15 × 10^9^/L within 2 days, accompanied by a flare of MAHA on peripheral smear. TPE was resumed, with the addition of rituximab on the first day and intravenous methylprednisolone pulse therapy.

Secondary causes were ruled out, including a negative full autoimmune panel, negative viral serology, and negative positron emission tomography (PET)-CT for malignancy.

Intravenous rituximab was continued weekly, and daily TPE was administered for another week until platelet counts exceeded 150 × 10^9^/L on two consecutive readings. After TPE, platelet counts continued to rise to 244 × 10^9^/L. She was discharged on tapering steroids, weekly rituximab, and aspirin, with close outpatient monitoring that included complete blood counts every other day. ADAMTS13 results later confirmed acquired TTP (aTTP) with low activity and a positive inhibitor.

The patient was readmitted 6 days later with a relapse, with platelet counts falling to 23 × 10^9^/L, hemoglobin of 10.1 g/dL, and hematocrit of 28.4%. TPE was resumed, and caplacizumab was requested as a lifesaving therapy.

During this relapse admission, the patient received 4 doses of rituximab, daily caplacizumab for 22 days, and 12 sessions of TPE. Platelets responded well with improvement in hemolysis markers. She remained stable with no further relapses and was discharged with a hemoglobin of 10.4 g/dL, hematocrit of 31%, and platelet count of 244 × 10^9^/L. Steroids were tapered gradually, and the central venous catheter was removed once the platelet counts remained stable. She made a complete recovery, with no recurrence over a 4-year follow-up period.

**Figure 2.**
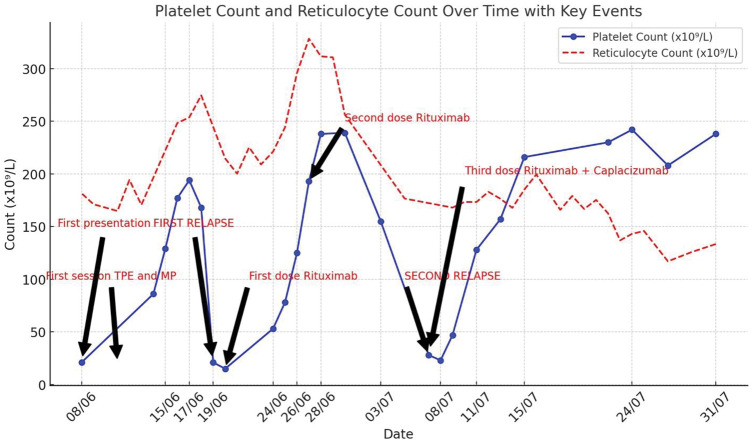
Case 3: Platelet and reticulocyte counts during the treatment period, showing fluctuations corresponding to therapeutic events, including TPE, rituximab, and caplacizumab administration. The graph highlights two relapses and the eventual stabilization of platelet levels following combination therapy. TPE: therapeutic plasma exchange.

Figure 2 illustrates platelet and reticulocyte counts over the period of treatment in case 3.

## Methods

The Case Report (CARE) checklist was followed during the preparation and writing of this manuscript.^10^

## Discussion

In this case series, three pregnant women presented with severe thrombocytopenia and MAHA, all ultimately diagnosed with TTP. Each case demonstrated characteristic features of TTP, including schistocytosis, anemia, elevated LDH, and negative DAT, with rapid improvement following TPE. The median gestational age at presentation was 33 weeks, consistent with the known third-trimester predominance of TTP.^3^ The cases highlight essential diagnostic challenges, including overlaps with other pregnancy-related TMAs such as HELLP syndrome, preeclampsia with severe features, DIC, and complement-mediated hemolytic uremic syndrome (cHUS)^4,5,11–13^ as well as the importance of early recognition and immediate initiation of TPE to prevent life-threatening complications. The heterogeneity in presentation emphasizes the need for high clinical suspicion, timely ADAMTS13 testing, and recognition of relapse risk, particularly in inherited cTTP.

TTP during pregnancy can occur as a new-onset condition or as a recurrence, triggered by physiological changes in pregnancy. Approximately 46% of TTP cases present in the third trimester, 38% between 20 and 29 weeks, and 15% before 20 weeks; when TMA appears in the first trimester, TTP is more likely.^8^ In aTTP, disease onset is typically sudden and severe, whereas cTTP often presents with isolated thrombocytopenia earlier in pregnancy, mimicking GT or ITP.^5,11^ Historically, diagnosis relied on a pentad—MAHA, thrombocytopenia, neurological and renal abnormalities, and fever—but only 10% of patients exhibit all five conditions. In practice, the presence of MAHA and thrombocytopenia warrants immediate evaluation for TTP and differentiation from other TMAs.^4,5,11–16^ TTP pathophysiology centers on severe ADAMTS13 deficiency (<10%), leading to accumulation of ultra-large vWF multimers and subsequent formation of platelet-rich microthrombi.^14,17^ Most acute cases involve autoantibody-mediated ADAMTS13 suppression,^16^ while cTTP results from pathogenic mutations in the ADAMTS13 gene.^12^ Pregnancy heightens susceptibility to TTP due to rising vWF levels and physiologic prothrombotic changes, particularly in the third trimester and early postpartum period.^12,16,18,19^

In our cases, thrombocytopenia was severe (9, 31, and 22 × 10^9^/L), and MAHA was confirmed by schistocytes (moderate in case 1, 3% in case 2, and 7% in case 3). Hemoglobin levels and LDH abnormalities reflected ongoing hemolysis and tissue ischemia but normalized after treatment.^15^ Negative DAT results excluded immune-mediated hemolysis, while normal prothrombin time ruled out DIC.^16^ Case 2 initially mimicked GT or ITP due to isolated thrombocytopenia, but rapid deterioration and red cell fragmentation clarified the diagnosis. Gestational ages (33, 31, and 37 + 4 weeks) and patient ages (median: 34 years) reflected typical disease patterns.^3^ Management emphasized prompt TPE initiation without waiting for ADAMTS13 results, given the high mortality risk if untreated.^5,8,20,21^ The cases’ differing ADAMTS13 and antibody profiles enabled classification: case 3 had aTTP, while cases 1 and 2 had cTTP. In case 1, sampling after TPE falsely elevated ADAMTS13 to 36%, underscoring the importance of obtaining samples before TPE to avoid diagnostic interference.^22^

Delivery was not used as a treatment modality, consistent with current evidence that delivery does not improve TTP unless indicated for maternal or fetal reasons.^23^ Failure of platelet recovery after several days of TPE should prompt evaluation for alternative TMA etiologies.^24^

TTP is a medical emergency, and daily TPE remains the cornerstone of management, reducing mortality from >90% to 10%–15%.^20^ Immunosuppression with corticosteroids is essential in aTTP to suppress ADAMTS13 autoantibody production.^8^ Rituximab, targeting B cells, is increasingly used to reduce relapse and hospitalization duration; it is administered weekly after TPE to avoid removal during exchange.^4,25^ Although rituximab may cause neonatal B cell depletion when used later in pregnancy, the benefits outweigh the risks in life-threatening maternal disease.^25–27^

Caplacizumab, a nanobody targeting the vWF A1 domain, prevents platelet–vWF binding and rapidly normalizes platelet counts.^28,29^ It is effective in refractory or relapsing cases, as illustrated by case 3, which achieved platelet stabilization and reduction of schistocytes after combined therapy with TPE, rituximab, and caplacizumab.^30,31^ Although pregnancy-related data are limited and the cost is high, caplacizumab may be lifesaving in severe or recurrent cases. Adjunctive low molecular weight heparin and low-dose aspirin may be considered when platelet counts exceed 50,000/µL, based on obstetric indication or thrombosis risk, although these are not routinely required.^5,12,20^

Neonates of mothers with cTTP require close monitoring for anemia, hemolysis, and jaundice, as severe cases may necessitate exchange transfusion; no complications occurred in our series.^32^ Long-term follow-up in future pregnancies involves monitoring of ADAMTS13 activity and hemolysis markers throughout gestation, with consideration of prophylactic plasma therapy in cTTP and delaying pregnancy for 6–12 months after rituximab in aTTP.^5^

Overall, this case series emphasizes the importance of early recognition of TTP, differentiation from other TMAs, and rapid initiation of TPE. ADAMTS13 testing, ideally performed before treatment, is essential for distinguishing aTTP from cTTP. Management of TTP is primarily similar in pregnant or nonpregnant patients, with some consideration for fetal safety when choosing immunosuppressive or targeted therapies. Emerging therapies such as caplacizumab provide additional benefit in severe, refractory, or relapsing disease. Multidisciplinary collaboration is essential to optimize maternal and fetal outcomes.

## Conclusion

TTP during pregnancy poses significant diagnostic and management challenges due to its rarity and life-threatening potential.

Early recognition, timely diagnosis, and prompt initiation of TPE are critical for improving both maternal and fetal outcomes. Although immediate access to ADAMTS13 testing is not available in all centers, TPE should be initiated if TTP is suspected, as delays may lead to serious complications.

Monoclonal antibodies, such as rituximab, play an important role in the management of aTTP, acting synergistically with TPE. Although caplacizumab is costly, it may be lifesaving in severe or recurrent relapses associated with high mortality risk.

This case series underscores the importance of interdisciplinary collaboration among obstetricians, hematologists, and critical care teams to ensure individualized management strategies for these complex cases.

## Data Availability

Data related to this case can be obtained by contacting the corresponding author.

## Declarations

This manuscript is original and has not been submitted or is under consideration for publication elsewhere. A preprint is available on Research Square.^33^ All authors have reviewed and approved the manuscript. All patient information has been fully de-identified.
